# Sensing Solutions for Collecting Spatio-Temporal Data for Wildlife Monitoring Applications: A Review

**DOI:** 10.3390/s130506054

**Published:** 2013-05-10

**Authors:** Mitra Baratchi, Nirvana Meratnia, Paul J. M. Havinga, Andrew K. Skidmore, Bert A. G. Toxopeus

**Affiliations:** 1 Persavive Systems Group, University of Twente, Enschede 7500 AE, The Netherlands; E-Mails: n.meratnia@utwente.nl (N.M.); p.j.m.havinga@utwente.nl (P.J.M.H.); 2 ITC, University of Twente, Enschede 7500 AE, The Netherlands; E-Mails: skidmore@itc.nl (A.K.S.); a.g.toxopeus@utwente.nl (B.A.G.T.)

**Keywords:** wildlife monitoring, spatio-temporal data, wireless sensor networks

## Abstract

Movement ecology is a field which places movement as a basis for understanding animal behavior. To realize this concept, ecologists rely on data collection technologies providing spatio-temporal data in order to analyze movement. Recently, wireless sensor networks have offered new opportunities for data collection from remote places through multi-hop communication and collaborative capability of the nodes. Several technologies can be used in such networks for sensing purposes and for collecting spatio-temporal data from animals. In this paper, we investigate and review technological solutions which can be used for collecting data for wildlife monitoring. Our aim is to provide an overview of different sensing technologies used for wildlife monitoring and to review their capabilities in terms of data they provide for modeling movement behavior of animals.

## 1. Introduction

Movement of individual animals plays and important role in different ecological processes [1]. This fact has laid the foundation of a scientific field known as “Movement Ecology”, which is a subset of ecological informatics, spatial ecology, computational geo-ecology, concerned with the ecological interactions associated with movement. As defined in Reference [1], movement ecology is a field that places movement itself as the basis, to promote the development of an integrative theory of organism movement to better understand the mechanisms, patterns, reasons, and effects of all movement phenomena. Examples of such phenomena in the domain of wildlife monitoring include animal resource selection [2], foraging [3], predation [4], and intersexual relationships [5]. Having spatio-temporal data of animal movement can further help in the design of animal controlling applications. For instance, to maintain landscape connectivity where animal migration paths intersect roads [6].

To realize the abovementioned ecological research, spatio-temporal data play an important role. One approach for collecting these data is through human observers. This is not always ideal as: (i) some animals are not easily observable as they are rare and nocturnal, tend to hide, or move fast and (ii) the presence of humans in the field is labor intensive, costly, disruptive to the habitat, and leaves negative impacts on the animal's health and behavior. Another form of data collection can be through using an automated system, which leaves no or little impact on the animal's behavior and health. Through such systems, data are collected with the help of sensors either directly attached to animals or deployed in the environment.

Wireless sensor networks provide the possibility of collecting fine-grained spatio-temporal data. These networks are known as the new generation of telemetry systems for wildlife monitoring. Various types of technologies can be used as sensors in such networks to collect different types of data. An important advantage of these networks over the previous telemetry systems is collaborative processing and data collection. In this paper, we review the sensing technologies used in such networks for ecological analysis. First, we classify the sensors used in two famous animal movement modeling approaches and then review data types that can be acquired using each of these sensors. Finally, we compare these sensing technologies in terms of their limitations, advantages, and data they can provide.

## 2. Classification of Technologies for Collecting Spatio-Temporal Data

**Figure 1.**
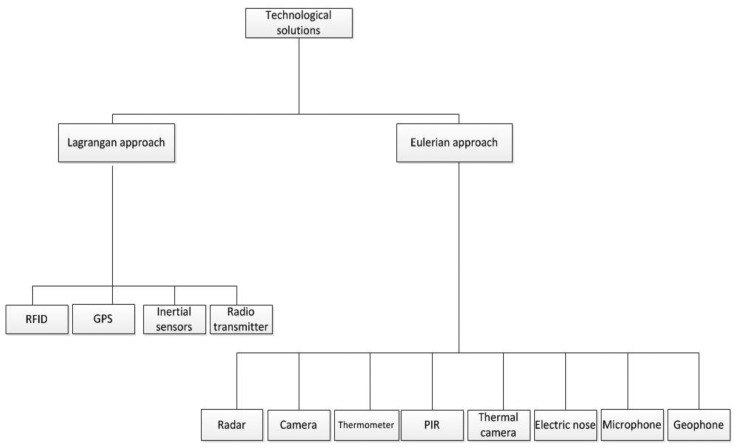
Taxonomy of the technologies used for collecting spatio-temporal data for ecological movement modeling.

Modeling animal movement from spatio-temporal data is generally performed using two approaches, *i.e.*, (i) the Lagrangian approach and (ii) the Eulerian approach [7]. The Lagrangian approach is individual-based and entails tracking a specific individual, while the Eulerian approach is place-based and deals with the probability of presence of an individual or a group in a place and the change of this occurrence over time. Motivated by these two approaches in modeling movement behavior from an ecological point of view, we classify and give examples of the technologies which can be used to collect data from animals in the rest of this paper. The general classification of these technologies is illustrated in Figure 1.

The essence of the Eulerian approach is modeling the pattern of space usage by an individual animal or a group of animals [7]. A suitable data collection method for such studies should be able to record the data from a point in space, and interpret events that occur in that point. The technologies used for realizing such a modeling approach should collect the data inconspicuously and in a manner that has least effect on the animal. This implies that these technologies may have more impact on the environment than on the animal, as their long-term placement and difficulty of retrieving them after use may have implications for the environment. The sensing devices of this type are deployed in the environment and process the disturbances caused by the animal's presence in the environment. Although it is more difficult to extract spatio-temporal data using these solutions, these technologies can provide more reliable results in terms of impacts they leave on the animal's behavior, and their health.

Various types of data can be retrieved from sensor technologies used for the Eulerian modeling without the need to trap animals. However, these data are subject to error and noise, and require extensive signal processing which is not always possible locally on the sensing devices. The technologies used for the Lagrangian modeling are, on the other hand, individual-based, invasive, in form of a mark or device which is fitted on the animals. They are designed specifically to retrieve specific spatio-temporal data type with high quality (plus, the identity data is directly implied because the device is fitted on a specific animal). However, accuracy is achieved at the expense of possible negative impacts on the animal's behavior or survival. These impacts may degrade the reliability of the data and should be studied and considered before analysis. There are also constraints in terms of the physiological properties, size, and weight of the animal which make marking and tagging hardly possible for some species.

## 3. Technologies for Eulerian Approach

As stated before, the technologies for Eulerian approach measure the environmental disturbances produced by the animals. The technologies which are used to detect these disturbances can be classified as passive and active [8]. Active detection technologies such as radar and sonar detect a target's presence by how it modifies an artificial sensing modality. Passive detection technologies simply record natural sensing modalities (visual, thermal, chemical, seismic, and acoustic) already present in the environment. In other words, active technologies both generate and receive sensing modality while passive technologies only receive a modality. From the technical point of view, three factors are of concern when designing a system for Eulerain modeling. These factors are: (i) choice of modality, (ii) technology and (iii) data analysis techniques to extract the spatio-temporal properties. A challenging task is data analysis since the measurements acquired by the sensing devices need to be further analyzed to extract information about the individual movement. In the following sections we review these modalities, type of sensors that can be used for each modality, and type of data that can be acquired using them considering wildlife monitoring applications.

### 3.1. Radar (Echoes Receptor/Generator)

#### 3.1.1. Echoes as a Modality

There are a number of animals such as bats and dolphins, which use echoes for sensing their environment. Bats design their waveforms considering that they want to classify by micro Doppler Effect (for dynamic targets such as insects or fish) or range profile information (for static targets such as flowers) [9].

Motivated by echolocation, active range Radio Detection And Ranging (Radar), Sound Navigation and Ranging (Sonar), and Light Detection And Ranging (Lidar) systems have been used for surveillance and target recognition and tracking. More recently, radar integrated with sensor networks has been found to be efficient when different categories of targets with individual identifiers exist.

#### 3.1.2. Technology

Radar systems are operable in different frequency bands. The best applicable band of operation for low power systems is the ultra-wide band (UWB). Instead of continuous narrowband transmissions, UWB radar systems use low-power impulses. This makes them suitable for sensor data collection and tracking applications. This type of radar is available in two groups, *i.e.*, (i) pulse Doppler, and (ii) pulse echo [10]. Pulse echo radar uses time of flight, applicable as rangefinder (detection of distance). Pulse Doppler radar, on the other hand, operates based on the Doppler principle (All moving objects will exhibit a frequency shift from the transmitted signal to the received signal which is proportional to the speed of the target in the direction of the radar), and is used for motion sensing (detecting location and velocity). There are currently radar sensors such as BumbleBee [11] compatible with wireless sensor boards commercially available.

While using radar to sense animal motions, the direction of motion can be critical in estimation of different parameters. However, by using a higher elevation angle one can avoid the issues experienced when the motion of animal is perpendicular to the radar's beam [12]. Doppler radar has the advantage of being able to directly collect measurements of an animal's moving parameters. It can be used for creation of a completely automatic activity recognition system. Based on the frequency/wavelength ranges, or “bands”, radar can penetrate barriers which obscure optical systems. For instance, UWB radar can be used in “see through the wall” applications. Other advantages of radar include operation in day or night operation, and over long distances.

#### 3.1.3. Data Analysis

Most previous research in collecting spatio-temporal data by radar work on the basis of micro-doppler effect. The velocity of a moving object relative to an observer can be estimated by measuring the frequency shift of waves radiated by the object. This is known as the Doppler Effect. In case of an articulated body such as a walking animal, the limbs and the trunk each have their own changing movements. The Micor-doppler effect is known as the modulation due to these movements [13]. The micro-Doppler effect provides signatures directly related to the dynamic structural parts of a moving animal and it offers new opportunities in classification of animals to different scales.

Various researches have been performed on extracting spatio-temporal properties from micro-Doppler effect. Examples include detection and classification of people when walking [14–16], finding the number of people present in the environment from their heartbeat patterns [17], distinguishing human from four legged animals [18], classification of different species by physiological characteristics [19]. Radar has been used to retrieve continuous spatio-temporal data on bird migration [20,21], and to retrieve birds' flying characteristics such as height, velocity, direction, and density regardless of the time of fly. Echoes have also been applied to identify different fish species at depth [22].

**Figure 2.**
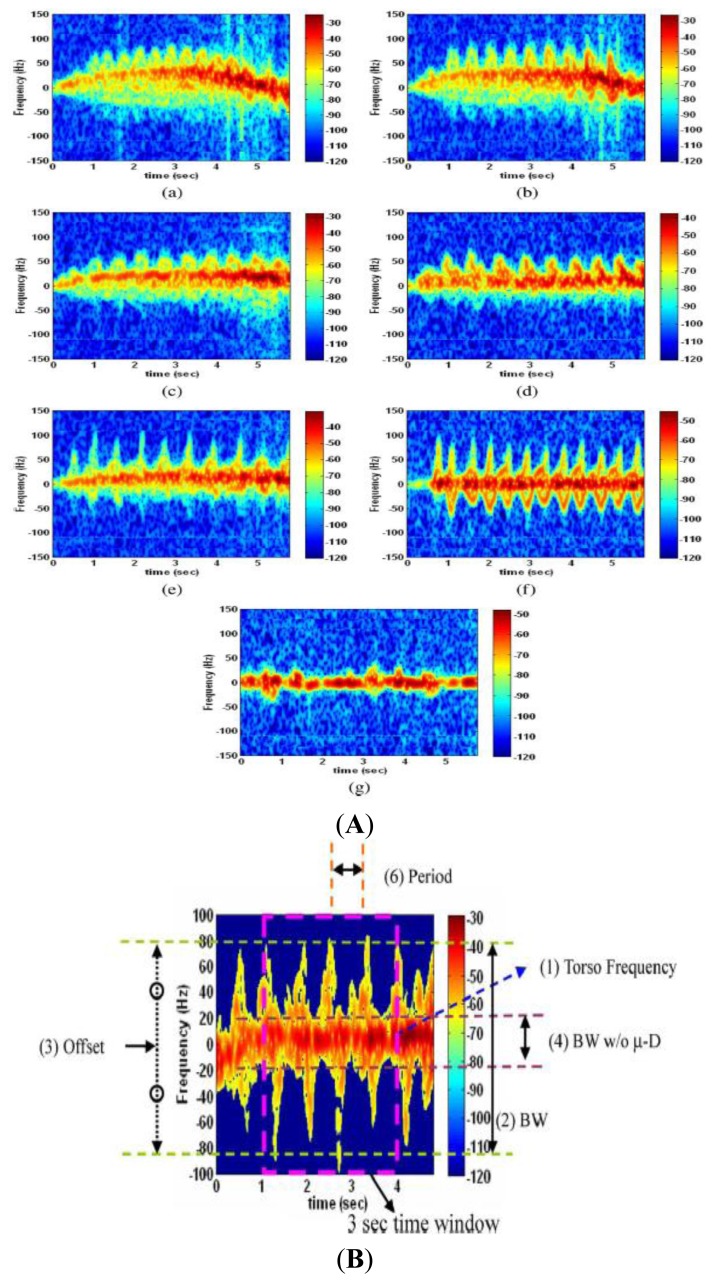
(**A**) Spectrogram of different human activities using micro Doppler signatures: Spectrograms of seven human activities. (**a**) Running, (**b**) Walking, (**c**) Walking while holding a stick, (**d**) Crawling, (**e**) Boxing while moving forward, (**f**) Boxing while standing in place, (**g**) Sitting with slight movements (© 2009 IEEE. Reprinted, with permission, from IEEE Transactions on Geoscience and Remote Sensing, 47 (5), pp. 1328–1337) [24]. (**B**) Different features useful in activity classification. (© 2009 IEEE. Reprinted, with permission, from IEEE Transactions on Geoscience and Remote Sensing, 47 (5), pp. 1328–1337) [24].

**Figure 3.**
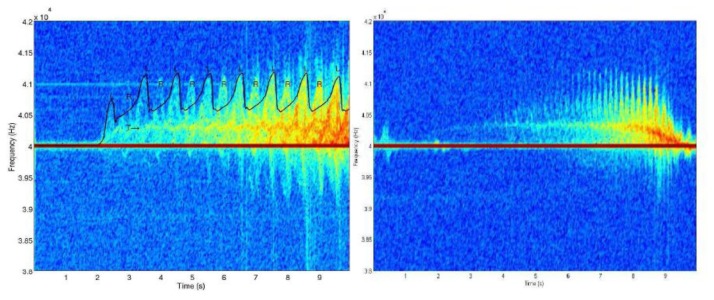
Micro-Doppler gait signature of (**Left**) a person; (**Right**) a dog walking towards an active sensing system. (© 2007 IEEE. Reprinted, with permission, from IEEE proceedings of 41st Annual Conference on Information Sciences and Systems, pp. 627–630) [18].

Other than identification and classification of animals at the species level, activities of the target can also be classified based on micro Doppler signatures. The subject of activity recognition by radar has been studied in [23,24] for humans and [25] for lab animals. Figure 2(A) shows the time varying Doppler signatures of a person while performing different activities. Combination of a number of features [24], such as period of micro Doppler, bandwidth, frequency, and torso Doppler frequency (shown in Figure 2(B)) in each activity is different. For example (as defined in Reference [24]), the micro-Doppler period in walking is longer than that of running. The torso Doppler of crawling motion is near zero and most of the micro-Dopplers are moving toward the positive values. Boxing while moving forward has a positive torso Doppler component and micro-Dopplers from the arms. Sitting has near zero torso frequency along with very small micro-Dopplers [24]. Figure 3 compares the spectrogram of a person and a dog. As can be seen, the period of the micro Doppler is clearly different between a human and a quadruped.

### 3.2. Cameras (Visual Receptors)

#### 3.2.1. Visual Modality

Visual interpretation of data can provide various types of information such as shape, size, and texture about objects. By extracting content information visually from a scene captured by a camera, different parameters, such as quantity, species of the animal, and movement characteristic can be detected.

#### 3.2.2. Technology

Integration of cameras and sensor networks has been studied in the context of multimedia and camera sensor networks. This has become possible with the availability of Complementary metal–oxide–semiconductor (CMOS) cameras. In comparison to previous charged coupled device (CCD) sensors, CMOS image sensors are smaller, lighter, and consume less power [26]. While regular CMOS sensors are still less energy efficient than the requirements of resource constraint applications, there is ongoing research to produce energy efficient hardware for use in camera sensor networks. Some examples of CMOS platforms are Cyclops [27], and CMUcam [28]. Hardware optimizations such as wake-up procedure, and dynamic voltage along with energy harvesting from the environment have made utilization of cameras in networks possible. Each of these smart camera nodes can process data locally and then exchange relevant information with other nodes. Furthermore, using a multi-tiered [29] network with different cameras (low/high resolution) in each tier can provide a more energy efficient system. In such cases, each camera can perform a different processing task with respect to its resources. One of the advantages of using normal cameras over technologies such as passive infrared sensors is their capability to identify cold blooded animals such as snakes. However, several factors such as foliage, lighting variations, and shadows can degrade the efficiency of camera based systems.

#### 3.2.3. Data Analysis

Visual data can provide effective identification mechanisms. Individual animals can be differentiated from each other through their visual differences such as biometrics. Various biometrics have been found in different animals (such as, iris patterns, skin ridge prints, tail patterns, and nose prints). Coat patterns present in many species (such as, cheetah, zebra, giraffe, orca, snakes) provide biometrics which are normally body sized and visible from distance. For example, automatic systems have been designed in Reference [30] to identify zebras based on their coat patterns. It should be noticed that in such systems other parameters such as angle of view and the change in natural marks (due to aging, injury, pregnancy) can also introduce false identification.

Other than natural markers, visual gait patterns can also be used to identify animals. Use of natural marks offers the advantage of detecting from long distances. Visual silhouette based gait recognition is extensively studied for human identification. The gait recognition can be performed through model-based or model-free approaches. In the model-based approach, the body and motion of moving organism is modeled through the use of a priori knowledge, while in the model-free approach gait appearance is considered without a priori knowledge about the underlying structure [31]. To identify species type, model-based approaches are more applicable as they consider body shape, while more precise identification of animals at the individual level requires model-free approaches. In wildlife monitoring, subjects such as classifying species [32] and identification of individual cows [33] through gait recognition of visual images have also been studied. A common characteristic in all these methods is that they consist of two main stages, *i.e.*, (i) a feature extraction stage and (ii) a recognition stage, in which standard pattern recognition techniques are used.

Vision-based localization methods can also be used to localize and track animals. Some previous studies have visually tracked flocks of birds [34], tracked animals using the gait patterns [35], and tracked lions by applying a specific model to the detected animal's face region [36]. Generally, target tracking with a single camera involves the following steps: target detection, classification, localization, and tracking from one frame to another. Selection of the right feature for tracking is an important step. Common features are color, optical flow, edges, and texture [37]. The vision-based localization which is performed by a single camera is limited to its field of view. In case more accurate result is needed, data from images of different cameras should be combined.

Images can also provide high amounts of information about animals' activities, behavior, and mutual interaction. Different methods, such as keeping the trajectory of the joints, action recognition with space-time volumes, or based on event and sub-events, can be used for the automatic single layer activity recognition [38]. Various vision-based activity recognition systems for wildlife have been designed. For instance, authors of [39] have designed a visual system to determine five basic behaviors, *i.e.*, sleeping, drinking, exploring, grooming, and eating of mice. In Reference [40] a system has been proposed for detecting snake behaviors such as attacking. A complete survey on human activity recognition can be found in Reference [38].

### 3.3. Thermal Sensors (Thermal Receptors)

#### 3.3.1. Thermal Modality

**Figure 4.**
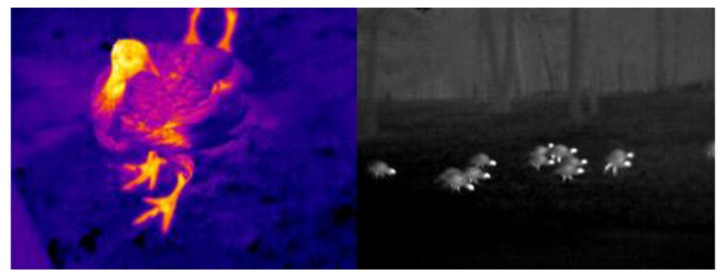
(**Right**) Infrared image of a Turkey, (**Left**) Detectable hotspots of turkeys for analyzing crowd behavior (Both images are provided by The Snell Group, http://www.thesnellgroup.com [41]).

All objects radiate infrared at normal room temperature. This electromagnetic radiation is a stream of photons, with no mass. Warm blooded animals such as mammals have considerably high amount of passive infrared radiation. Thermal hot spots in these animals can distinguish them from vegetation, and enable a means for detecting them. As Figure 4 suggests, factors such as the number of animals or the species is to some extent detectable visually using thermal sensors from the hotspots.

#### 3.3.2. Technology

Three types of sensors can be used to detect infrared radiations from animals. These sensors are (i) thermal imagers, (ii) passive infrared motion (PIR) detectors, and (iii) passive infrared thermometers.

Thermal imagers work on the basis of Infrared Thermography (IRT) which is the measurement of radiated electromagnetic energy. There are some limitations and factors that need to be considered when getting thermal images. Heavy feathers and furs will reduce detectable radiation. The hair which covers the animal should be free of dirt and moisture, since the dirt on the animal changes the emissivity while moisture increases local heat loss [42]. To benefit from infrared radiation in low-powered networks, PIR motion detectors and passive infrared thermometers can be used. PIR motion detectors are devices which detect motion in their field of view by measuring changes in the infrared radiation from their surrounding objects. Due to their low cost and low operation power, these sensors are popularly used in wireless sensor networks for the surveillance purposes. Available wireless sensor node for ecological applications, are already equipped with passive infrared motion detectors as well as other environmental sensors (such as, the Mica weather board) [43]. Passive infrared motion detectors have been popularly used in combination with cameras in form of camera traps [44]. As shown in Reference [45], strength of the output signal of the PIR sensor is not only determined by distance but also by speed of the moving object. Therefore, a PIR sensor network and simple signal processing algorithms can be used to obtain parameters needed for wildlife activity monitoring (in the covered area) such as direction, speed, distance, and counting the animals. A disadvantage of PIR sensors is that they can only detect presence in motion and presence of a static subject is not detected by them.

#### 3.3.3. Data Analysis

Since most warm-blooded animals have similar temperature ranges, the thermal signatures will not accurately discriminate between species. However, they can show temperature-related states of the animals. As mentioned before, PIR sensors are widely used as presence detectors. Infrared cameras can provide information on specific species if the species has a discriminative shape (for instance, humans have been detected based on thermal shape [46]). Research has been performed in identification of individual human beings based on their thermal information (such as, face recognition by capturing facial physiological patterns using the bio-heat information extracted from thermal images [47]). By considering and counting the hotspots in a thermal image the crowd behavior can also be analyzed [48]. Infrared thermography combined with infrared cameras has been used to detect physiological states in humans and animals. In Reference [42], infrared thermography is used to measure the stress level in farm cattle. The basis of this research is that when the animal is stressed, changes will happen in heat production and heat loss in addition to blood flow response. Processing thermographic images can provide information about the animal's stress level, health (such as, asymmetric heat distribution [49], abnormal surface temperature [50]), pregnancy, and any property related to the normal body temperature change.

### 3.4. Chemical Sensors (Chemical Receptors)

#### 3.4.1. Chemical Modality

All living creatures produce volatile compounds. Different environmental, genetic, and dietary circumstances, makes it improbable that any two organisms produce the same mixture of volatile organic compounds. On this basis, many animals can identify the members of their own group in a large group of other individuals [51].

#### 3.4.2. Technology

**Figure 5.**
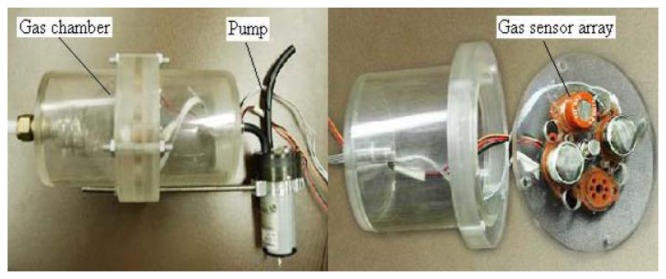
An electronic sensor node designed for the purpose of monitoring odorant gases and accurately estimating odor strength in and around livestock farms (Reproduced with permission from Simon X. Yang, Sensors, published by MDPI, 2009) [57].

Electronic noses have so far been used in various fields such as agricultural, biomedical, environmental, nutrition, medical, and military (A prototype is shown in Figure 5). Each electronic nose has two functions, *i.e.*, (i) sensing, and (ii) pattern recognition [52]. For sensing, chemical and gas sensors are used. By using a sensor array composed of different sensors, a wide group of simple and complex compounds can be identified. Different sensors have been used in the past to detect odors such as piezoelectric, conductivity, metal-oxide-silicon field effect transistor (MOSFET), optical fiber, and MEMS based sensors. Various types of artificial intelligence methods can be used for the purpose of pattern recognition to identify the smell [52].

#### 3.4.3. Data Analysis

Chemical cues are important phenomena in the biology of animals. Sex pheromones are the well-known examples of chemical communication in different species. Producing electronic noses for detecting pheromones has been explored for insects. Previously, biosensors have been produced by real moth antennas to extract electrical signals which are produced in existence of other moths [53]. Pheromone detection of insects along with their specification such as gender and species from their pheromones is studied in Reference [54]. In Reference [55], commercially available electronic noses (such as, the Cyranose [56]) are used to detect stink bugs and their damage in cotton product under laboratory and field conditions.

One of the important issues in designing an electronic nose is that the combination of volatile compounds of the odors should be detected beforehand. This procedure is normally done through gas chromatography and mass spectrometry [58]. It should be noticed that for typical sensors, the detection threshold of odors are extremely low. Therefore, detection of a very low amount of gas compound in a large environment is challenging. To be used around livestock farms where the volatile organic compounds are well known, commercially available gas sensors and high intensity of gas make the compounds easily detectable [59]. However, although detection, tracking, and identification of specific animals (especially mammals) is possible, extensive research should still be performed on pre-analysis of the compounds.

Electronic noses have been used to identify human beings from their body odor [60]. Although individuality in body odors has been described in a variety of mammals [61], the electronic noses which have been designed so far for animal studies mainly focus on purposes such as controlling gas level around farms other than identification. Moreover, the changes in the individual compounds due to effects of physiological and seasonal changes should be considered for designing a system for classification [62].

Since the composition of the compounds in animal's odors change with various physiological parameters (such as, their health condition, age, and estrous cycle of the animal), the electronic noses can be used to detect events that cause changes in the compounds. In such cases, placement of electronic noses in places with rich sources of known volatile organic compounds is more logical. For instance, in Reference [63] e-noses show a correlation between evolution of the odor with animal activities during the day and with their age around the farm.

### 3.5. Microphones (Acoustic Receptors)

#### 3.5.1. Acoustic Modality

Animals use vocal sounds for different purposes such as defending their territories, attracting opposite sex, deterring predators, contacting with members of their social group, and navigation [64]. Animal sound production capability can be divided into two categories, *i.e.*, (i) non-incidental sounds which are used for communication purposes, and (ii) the incidental ones which are the result of their activities. These sounds can be used to detect the presence or to identify a species. An important challenge in acoustic sensing is that ambient noise and anthropogenic sounds can make the acoustic signal processing difficult.

#### 3.5.2. Technology

Microphones are acoustic transducers which produce a voltage proportional to the received acoustic pressure. Microphones work at different frequency ranges and the right frequency of operation is chosen by considering the type of sound produced by animals. Generally, animal voices are categorized as sonic, infrasonic (elephants), and ultrasonic (bats and dolphins). This fact determines the type of microphone needed to capture the sound of interest. Directional microphones can be used for capturing sound from specific directions [64]. Due to extensive amount of research done in the field of acoustic sensor networks, microphones are already part of commercial wireless sensor network boards (such as, the Mica sensor board [65]). Furthermore, specifically designed platforms for acoustic sensing networks are available. For instance, the Acoustic ENSBox system provides a platform for distributed acoustic sensing and source localization applications [66].

#### 3.5.3. Data Analysis

Different algorithms allows to distinguish animals in terms of species, gender, age groups, and individuals by automated signal detection and classification based on features extracted from signal frequency, mel-frequency cepstral coefficients, or signal energy distribution [64]. Both voiceprints and behavioral sounds can be used for the purpose of classification of animals in different levels. Regarding animal voice recognition, most of the researches focus on species identification [67–69] and specifically on classification of bird species based on their song [70,71]. Individually distinctive acoustic features have been demonstrated for a large number of birds, mammals, cetacean, and amphibians. Various studies have been performed to identify individuals based on these voiceprints (whooping cranes [72], African wild dogs [73], eagle owls [74] and ant-thrushes [75]).

Other than non-incidental voiceprints, incidental sounds may entail information about the identity of the species. Although to the best of our knowledge, there is no work on classification of animals based on their acoustic gait signature, footsteps sound analysis has been used for human detection [76] may be applicable for animals. Studies show that footstep convey information about personality, age, and gender [77].

An animal's position can be determined using an acoustic localization algorithm. Sound signals are omni-directional and have a uniform attenuation model. Microphone arrays can be used to provide efficient localization of the animal without having a line of sight to the animal which produces the sound. Through localizing animals acoustically, information about their interaction, count, and population distribution patterns can be extracted [64]. In localization applications several sensor array nodes need to be located in the animal's territory and when detectable sound is made, the position will be estimated from sensor measurements. Acoustic source localization methods normally make use of three different types of physical measurements, *i.e.*, time delay of arrival (TDOA), direction of arrival (DOA), and received signal strength or energy.

### 3.6. Seismic Sensors (Seismic Receptors)

#### 3.6.1. Seismic Modality

Producing seismic vibrations on a substrate is a means of communication in different species (invertebrate and vertebrate). An interesting seismic effect produced by legged animals is footsteps. Animals have distinctive gait patterns (4-beat gait, 2-beat gait, and canter; 3-beat gait and some unnatural walking patterns, collective walk or working walk [78]). Footsteps produce seismic effects that pass through the ground. These effects propagate away from the source as seismic waves. These waves are classified into two categories, *i.e.*, (i) body waves (33%), which travel towards the interior of the earth, and (ii) surface (Rayleigh) waves (67%), which travel near the surface [79]. Most of the researches focus on surface waves to provide classification systems of targets.

A number of factors determine the performance of vibration detection. These factors include the resonance frequency of a vibration, the frequency of impacts (footsteps), the strength of the wave (enforced by animal weight), the friction of the medium, the underlying geology [80] and noise sources such as wind and cultural noise (undesirable noise produced by human urban activities). Wind noise may be coupled into seismic ground sensors by direct or indirect (adding seismic noise through shaking trees) means [80].

#### 3.6.2. Technology

Surface waves are measured by two types of transducers; (i) geophones, and (ii) accelerometers. Geophone is a device that changes the velocity of ground vibrations into a voltage. Geophones are normally buried to keep them safe from animal's destruction. As stated in [80], these devices show both low-frequency (10 Hz, 14 Hz, 28 Hz, and 40 Hz) fundamental resonance and high-frequency spurious resonance (25 times of the fundamental). For detection of vibration generally only frequencies that lie between the fundamental and spurious resonances should be used [80]. Most energy of human footsteps is between 10 and 100 Hz repeating with a frequency between 0.9–3.5 Hz [81]. As the authors of [82] have characterized, the main part of the footstep signal energy for a distance more than 6 meters, is usually bellow 100 Hz. These researchers have shown that, as the distance between a walking person and a seismic sensor increases from 6 to 60 meters, the signal frequency maximum moves closer to 10–16 Hz. The resonant frequency range of the animal footfall and these facts should be employed in choosing the right geophones sensor. The second type of seismic sensors is accelerometers, *i.e.*, a device that change the acceleration of the ground vibrations into a voltage. Accelerometers only show high-frequency resonant frequency (over 1,000 Hz [80]) and this makes them unpopular in footstep detection. However, when the acoustic waves are transferred through the substrate, these vibrations can be detected with an accelerometer. For instance, high-frequency acoustic waves can also be detected with accelerometers while passing the substrate [83]. Accelerometers have also been part of the Lagrangian wireless sensor network systems to collect movement data from animals [84].

#### 3.6.3. Data Analysis

Numerous seismic surveillance studies have been performed to classify targets such as, vehicles and soldiers, compute their bearing, and their velocity based on seismic features [85]. Humans and animals can also be detected based on their footstep-generated seismic waves. The signature of footstep is in form of sharp “spikes” and distinguishable from other noises [86]. By measuring the seismic signals using a seismic velocity transducer presence of the moving human or animal can be detected. Different features and statistic characteristics of signal can be used for detection and distinguishing the animal. Afterwards, the signals can be classified using artificial intelligence methods. For example, spectral analysis for discriminating between seismic events caused by animal's footsteps, cadence [78] (the interval between events (footsteps)) and kurtosis [87] (degree of peakedness of a distribution) are used for footstep detection. In the domain of human sensing, seismic waves have been used to detect presence of humans [88,89], tracking and bearing estimation [90]. Mainly the seismic studies that consider animals have focused on differentiating between two categories (bipedal (human) and quadruped (animals) [78,91]). Few works on pure wildlife studies have focused on problems such as detection or classification of animals. For instance, [92] has investigated a number of problems, such as detection of elephants from a distance of 100 meters, counting the number of individuals, and differentiating their species from other species. The species (to some extent) might also be detected based on the influence field (the number of sensors that sense the vibration [8]). Seismic communication, foot-drumming, distinctiveness of footsteps in terms of gender of animal have also been studied [93]. Moreover, in low-noise environments underground organisms can be detected. For instance, in Reference [83] the acoustic waves produced by a colony of ants underground is detected and classified by geophone from a distance of few centimeters.

## 4. Technologies for the Lagrangian Approach

As stated before, the Lagrangian based technologies are in form of a tag or device which is attached to the animal. Generally, when choosing an Lagrangian based technology some general requirements need to be kept in mind:

- The device should preferably weight less than 3–5% of the animal's total bodyweight (no more than 10% for terrestrial mammals [35]).
- The device should have a relatively long lifetime so that it is not needed to trap the animal again before the necessary amount of data is collected.

Compared to the previous group of technologies, *i.e.*, technologies for Eulerian approach, the issues that should be greatly concerned in Lagrangian approaches are the choice of the hardware and retrieval of data from the tagged animal. When used on wild animals, it is important to have a mechanism for automatic retrieval of the data from the tag through single/multi hop networks as the chance of recapturing the animal is small.

In what follows, we review a number of technological solutions used in the Lagrangian approach for collecting spatio-temporal data for wildlife monitoring applications and their integration with wireless sensor networks.

### 4.1. Radio Frequency Identification

#### 4.1.1. Technology

Radio Frequency Identification (RFID) is a technology designed for storing and retrieving data by using electromagnetic transmission. Nowadays RFID is being used as a means of enhancing data handling tasks [94]. The RFID systems consist of two main components, *i.e.*, tags and readers. Each tag has a memory that stores an Identification number. This memory can also store additional data such as environmental parameters (temperature, and humidity). The reader (including an antenna) reads and/or writes data to tags through electromagnetic transmissions. RFID tags have been used to study various species (birds [95], reptiles [96], amphibians [97], mammals [98,99], and humans [100]).

RFID technology is originally designed for retrieving identity but it can be used to retrieve location as well. After detecting and identifying the moving tag, different types of algorithms can be used to calculate the current location of the tag, relative to the readers' location. Localizing techniques for RFID tags are known as RF based localization which lie on the same principles of the ones for wireless networks [101]. To save power in an event-based manner, the procedure of tracking the object can be done in a predictive manner to activate the readers which are in idle state.

**Figure 6.**
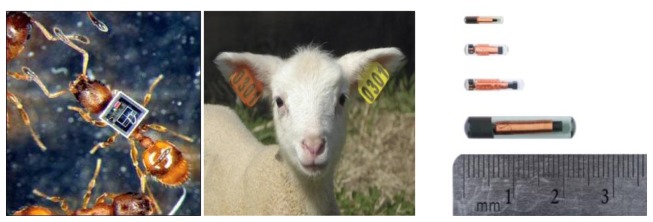
(**Left**) A radio tagged ant (Reproduced with permission from RFID Journal, 1 August 2009. Image, and copyright held, by, Nigel R. Franks) [107], (**Middle**) Passive RFID ear tags (Image provided by Premier1Supplies [108]), (**Right**) Passive RFID implants (Image provided by Biomark Inc. [109]).

- *Tags*: RFID tags themselves may be active, passive, or semi-passive. They may be supplied in a variety of forms and work based on ISO standards [102].
  - *Active Tags*: Active RFID tags are equipped with their own independent power source. Thus, they are able to transmit a stronger signal which can be accessed by readers placed in far distances. These tags operate at higher frequencies, commonly 455 MHz, 2.45 GHz, or 5.8 GHz. Based on the frequency of operation, readers can communicate with active RFID tags from a distance of 20 to 100 meters [103]. The onboard power source makes the active tags larger and more expensive, mainly practical in terms of monitoring large animals. They are normally carried around by animals in form of collars or harnesses. The lifetime of tags can be increased by sleeping schedules which activate the tags as they come in range of a receiver.
  - *Passive tags (Passive integrated transponder, PIT tags)*: For wildlife monitoring purposes passive tags are made available in different forms (implants, or ear tags). They consist of three parts, *i.e.*, (i) an antenna, (ii) a chip attached to the antenna, and (iii) encapsulation. Passive tags do not have an internal power source and the reader is responsible for powering them. When these tags come within the reader's range, they receive an electromagnetic signal from the reader, and the energy is stored in an on-board capacitor. Because of their small size and weight, they are useful to study small animal movements with less disruption. Furthermore, without having a power supply they will last for the life of the animal. This technology is very popular for tagging fish [104] and it has even been used for studying ants [105]. Passive tags normally work at frequencies of 128 kHz, 13.6 MHz, 915 MHz, or 2.45 GHz and can be read in ranges between a few centimeters to 10 meters [103]. Factors such as frequency of operation, antenna dimensions, and modulation type determine the read range [106]. Since the water in living tissues absorbs high frequency photons, most of the passive implants designed for identifying animals operate in low frequency [106] (125-kHz and 134.2-kHz), while passive external tags work in higher frequency ranges. Passive tags are small and cheap themselves but the readers are relatively big and noticeable and for having better detection range, the size of the antenna increases extensively. Figure 6 shows a number of passive RFID tags used for monitoring live organisms.

#### 4.1.2. Integrating RFID Technology with Wireless Sensor Networks

Traditional RFID technologies work on the basis of single hop communication between a reader and a tag. Integration of RFID readers within a sensor networks has improved functionalities of these systems both by enjoying the capabilities of wireless communication and retrieving additional data [110]. In this type of integration, the RFID readers are provided with multi-hop communication capability and other type of sensors. They will be able to communicate with each other wirelessly and sense environmental parameters and disturbances. This type of integration is an ideal solution for collecting various types of data through one network for the Lagrangian approach. The high quality identity information is achieved by the readers and other types of data such as environmental data and activity information are collected by the sensors (For instance, a camera) on the RFID reader and are then transferred through a multi-hop fashion. The SkyeRead Mini M1 made by SkyeTek is an example of an RFID reader (for reading 13.56 MHz RFID tags) designed to mate directly with the Crossbow Mica2Dot sensor mote [111]. This kind of RFID reader can be used with various types of passive ear tags. In Reference [98] a hybrid detection node is designed by integrating RFID readers with Tmote Sky motes for collecting spatio-temporal data from badgers. Authors of [99] have interfaced the tag readers with Fleck wireless sensor network nodes to track the movement of farm animals near the readers.

### 4.2. Global Positioning System (GPS) Technology

#### 4.2.1. Technology

**Figure 7.**
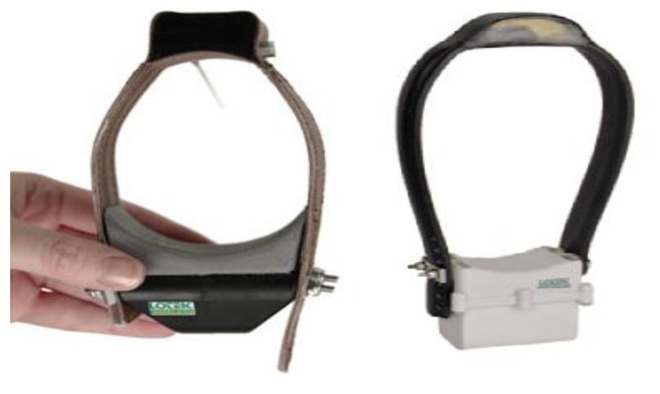
GPS collars: (**Left**), GPS collar designed for small mammals, (**Right**) GPS collar designed from medium-large sized mammals (Images provided by Lotek Wireless Inc. [118]).

Global Positioning System (GPS) is a widely used localization system in various domains and specially in wildlife monitoring [112,113]. GPS devices can be considered as invasive sensors for position sensing and localization. The system components are a space segment (24 satellites), a control segment (network of ground based stations) and a user segment (receivers that convert satellite signals into location estimates) [114]. The receiver acquires signals from at least three satellites to obtain 2D positions (four satellites for getting 3D positions). There are two approaches to retrieve the data collected by the user segments, *i.e.*, offline and online. In the offline approach, the user segment should store the data on board, which will be later either retrieved after the user segment is collected or manually retrieved by handheld receivers. To access the data in real-time, different telemetry systems such as the ARGOS satellite system, radio telemetry transmitters, and GPRS have been used so far. Recently wireless sensor networks have been used to transfer GPS data as well. It should be mentioned that due to energy consumption and size restrictions, currently, it is inevitable to have the data collected offline in certain cases otherwise the system will not be able to meet the average life-time requirement. Today, several commercial GPS equipment such as Telemetry Solutions [115], Televilt [116], Northstar [117], Lotek [118], E-obs [119], Microwave [120] are available, which provide more than just positional data. These devices are designed for almost any animal (birds, mammals, reptiles, and rodents) and depending on their size and weight, these GPS receivers can be equipped with bidirectional transmitters (work sometimes up to 400 meters), a set of sensors, contact loggers (to log the contact between two animals) and a power source (two GPS devices are shown in Figure 7). Also, having considered wireless sensor networks requirements, Fleck family boards [121] have been designed for the wildlife monitoring purposes. GPS is designed for localization. However, since the GPS is attached to an animal, the identity is provided by an ID sent with every position. Other than the risks that it may have on the animal's health and normal activities, another limitation of the GPS devices is that in certain conditions the GPS receiver will not be able to receive enough satellite transmissions. These conditions include: (i) atmospheric conditions like cloud cover, humidity, (ii) biophysical conditions like under dense foliage, steep terrain or buildings, (iii) indoor applications (farm animals), and (iv) changes in the orientation of the antenna due to animal behavior). By later analysis of GPS data, various interesting patterns can be found [122].

#### 4.2.2. Integrating GPS Technology and Wireless Sensor Networks

When using the GPS as sensors of wireless sensor networks, generally two types of communication architecture will be possible:

- *Mobile node to mobile node communication*: In this type of communication, mobile wireless sensor nodes fitted on animals are equipped with GPS modules. These mobile nodes are the only constructors of the system and the data they collect should be passed from a mobile node to another mobile node until it reaches the gateway. In this case, the advantage that wireless sensor networks have over the previous telemetry mechanisms is providing the capability to extend the transmission range and reduce power consumption of the GPS device through opportunistic routing protocols. In this form of architecture, if none of the animals (GPS receivers) comes to the proximity of the gateway, no data is transferred. So far, various wildlife studies have transferred GPS data with the help of wireless sensor networks with this architecture [123–126].
- *Mobile node to static node*: In the second type of communication, other than the mobile nodes which are equipped with GPS modules, there is a ground based wireless sensor network that collects data from the mobile nodes. This form of communication alleviates the problem of sporadic connectivity of the previous one. In Reference [127], authors have designed a system in which GPS devices fitted on animals are able to communicate with an array of static nodes to return data to a central base station.

In both of these type of communications, the system can be devised to be dely-tolerant which means that data can be buffered in a node before it comes to the proximity of another node. However, they have a lack of applicability when animals travel far distances and do not return to the connected area.

### 4.3. Inertial Sensors

#### 4.3.1. Technology

By using inertial measurements such as speed and direction, the track of the moving animal can be estimated. Inertial measurement unit sensors such as accelerometers, gyroscopes, and magnetometers can be used for this purpose. Accelerometers, gyroscope, and magnetometer measure acceleration, angular velocity and magnetic field respectively. Metal objects, external magnetic field and gravity are sources of error in inertial sensors.

By knowing the animal's initial location, its next position can be estimated having the velocity or acceleration during that time interval. This scheme is referred to as Dead Reckoning and it is a complementary addition to GPS systems for preserving energy and retrieval of localization data when a GPS device is not responsive (due to cloud cover). However, this method is subject to accumulative error. Dead reckoning has been used frequently for indoor tracking of human and pedestrian navigation [128,129].

#### 4.3.2. Integration with Wireless Sensor Networks

If inertial sensors are embedded on sensor network nodes attached to animals, they can be used for the purpose of localization. For instance, authors of [130,131] have designed a system for localization of rats in underground burrows to estimate their steps with inertial sensors. On the exit of the burrow the data (the number of steps and their direction) is transferred to a static node, through which the path and structure of the underground borrow is constructed. This form of tracking, although not very accurate, can be effective when a network of static nodes is used to update the position of the mobile sensor nodes. Inertial sensors have also been popularly used in activity recognition and behavior analysis systems for humans [132] and animals [84,133,134].

### 4.4. Radio Transmitters

#### 4.4.1. Technology

Using radio transmitters for wildlife tracking has long been popular. Radio telemetry systems consisted of a device attached to the animal which transmitted a radio signal. The position of the animal could be retrieved by triangulation. Later some additional sensors were added to those devices and additional parameters could also be sent through radio telemetry. The tracking range in radio telemetry methods is limited by radio range of the UHF (ultra-high frequency) or VHF (very-high frequency) and the devices used are relatively power hungry.

#### 4.4.2. Integration with Wireless Sensor Networks

Recently more efficient systems have been designed using wireless sensor networks. The wireless motes consisting of a transceiver, memory, and a micro-processor (no other sensors) are fitted on animals. By doing so, the position of the animal is estimated through radio communication between these mobile nodes and a static gateway, without the help of any additional sensors. For instance, in Reference [135] animals register their presence when they come into the transmission range of a gateway and the received signal strength is used at the gateway to estimate the location of the animal. This form of localization and identity retrieval is in essence a better alternative than using active RFID tags, which register the presence of the tagged animal near the reader. In this case there is no need to interface a commercially designed reader with the gateway (*i.e.*, a wireless sensor network node).

## 5. Discussion

In this section we compare the aforementioned technologies in terms of data they provide and their applicability for wildlife monitoring studies. Tables 1, 2 and 3 provide a comparison of the technological solutions mentioned previously from different perspective. By focusing on movement ecology we only compare the aforementioned sensing technologies in terms of their spatio-temporal data collection capability and do not consider other data types such as behavioral and physiological data. Adopting the definition of spatio-temporal properties in the domain of human sensing [136], we identify and define track, identify, species type, location, presence, and time as spatio-temporal features needed for movement ecology.

- *Track*: is the most comprehensive spatio-temporal feature, which can be simplified as the location of an individual or a group over time. Therefore, the three features of location, time, and individual/group identity are needed to maintain track of an individual or a group.
- *Identity*: is a global unique identifier assigned to an animal. It may be the permanent ID of a tag, or a detectable biometric or sign which can show the individual identity of an animal. When assigning a unique ID to an animal is not possible, a temporary identifier may be used.
- *Species type*: The species type can be considered as a low level identity that can be assigned to members of a species. We can also consider it as a sub-property of identity.
- *Location*: Localization is determining the location of an animal. Whenever coarse grained coordinates are acceptable, localization can be reduced to presence detection. Otherwise, a separate mechanism should be used to localize to a finer grain scale. For example, a single microphone can be used for detecting the presence of an organism through its voice but it is not enough to detect its location, the position which can be inferred in this way is only an estimate around where the microphone is placed which might be a relatively large area. For having more exact location coordinates a microphone array is needed and the fine coordinate can be calculated through various acoustic source localization schemes.
- *Presence*: Detecting presence is the ability to detect the presence of an organism in a field. Detection alone without classification of the species, detecting the identity, or the number of subjects may only provide limited amount of information. However, technologies that can only detect presence of an animal can be used as an input to other more powerful but yet more resource consuming technologies.
- *Time*: Time is an essential property among spatio-temporal features. There should be possibility of assigning timestamps to the features which are detected to make them meaningful for later analysis. The frequency of timestamps is relative to how frequent other features should be sensed so that the integrity of data is not lost.

### 5.1. Comparison of Technologies Based on Information They can Provide

In Table 1 we compare the previously reviewed sensors in terms of their ability to provide the aforementioned spatio-temporal features. The field under a feature is marked with a (✓) if the results of previous researches show that this technology is applicable for the purpose of obtaining spatio-temporal features in wildlife monitoring applications. Otherwise, it is marked with (-).

Most of the technologies which we identified for the Eulerian approach can detect presence. If the purpose is to detect the presence of a warm-blooded animal, PIR sensors are particularly useful. Radar, geophones, and microphones are capable to extract all above-mentioned spatio-temporal features. By performing image processing techniques on the images collected by thermal cameras some spatio-temporal features can be extracted. Since most of the visual biometrics are not shown in thermal images, the applicability of thermal cameras in identification is low. As mentioned above, electric noses are theoretically able to identify individual/species but the plausibility of current electric noses is not yet proved for wildlife monitoring approaches (due to the low amount of organic compounds).

The Lagrangian based technologies are more useful in extracting spatio-temporal features from individuals. RFID tags work on the basis of transmitting an ID number. The devices which are equipped with GPS, radio transmitters, and inertial sensors can be programmed to send a unique ID number periodically. Being able to extract the individual identity, presence and species type of the tagged individual can also be inferred. Therefore, all these technologies are marked with + indicating their ability to provide presence, species type, identity, and location information. Although RFID tags and radio transmitters are not designed to measure location, location information can be calculated from specifications of the signal transmitted between the device on the animal and readers (or receiver). A single device which is equipped with GPS and inertial sensors estimates location and there is no need to calculate the location by taking the measurement of other devices into account.

The major difference between technologies used by the Langrangian and Eulerain approaches is the applicability of the Eulerian based technologies for extracting spatio-temporal features from any animal in their field of view, while technologies used by the Lagrangian approaches are only useful for extracting these features from the tagged animals.

Track is the only spatio-temporal feature which is not mentioned in Table 1, because it has to be inferred from combination of other features. For instance, a network, which can continuously detect presence of an animal, or a sparse network with identification capability, can both provide tracks. Choosing which spatio-temporal features to use for tracking depends on the data needed for the purpose of later analysis. For instance, for monitoring migration of specific species, species recognition with coarse grained localization is enough to provide the necessary tracks since individuals stay in the flock. Therefore, sometimes the group track might be more important than the individual track.

### 5.2. Comparison of Technologies Based on Different Performance Metrics

By reviewing previous research, a number of general conclusions can be drawn which can help researchers in choosing an appropriate sensing technology. Regarding the power consumption, passive and active mode of operation impacts the energy efficiency. Active mode of operation requires the generation of a sensing modality and is, therefore, more energy consuming. Some passive technologies such as visual and thermal cameras are also not energy efficient since the visual data type is considerably large, and requires more memory and processing power.

Among the sensors used in the Eulerian approach, PIR is known to be the most energy efficient, and low cost. It is widely used in low power surveillance systems where the area is covered with a large number of nodes with short coverage range. However, the information which can be extracted from these sensors is limited. Camera, microphones, and radar are more suitable for longer range operations depending on the characteristic of the target. These sensors also provide more information from the studied animal. It is also important to consider the line of sight requirement of the sensor to the animal. Among these technologies, thermal and ordinary cameras, PIR and thermometers need a direct line of sight to the target. Based on the frequency of operation radar can be used when physical barriers exist between the sensor and the target.

In Lagrangian based approaches, use of GPS is specifically recommended for long range localization purposes due to its global coverage and accuracy (relative to the covered area). However, GPS is considerably power hungry. In order to meet long lifetime requirements, it is sensible to use duty cycling and low power inertial sensors to measure the approximate location when the GPS module is off. The shortcoming of the technology relates to the fact that its applicability has been only proven for heavy animals as a GSP-enabled device with a lifetime of few days/weeks is relatively heavy. Active RFID tags and short range radio transmitters are more useful when the weight requirements are critical and for animals with limited range of spatial activity. Although passive RFID tags are small and light, their extremely low detection range limits usage in an automated system. Furthermore, when using RFID tags energy provision for an automated reader remains a challenge.

In Table 2, we compare the technologies in terms of the: (i) outdoor disruptions that degrade the performance of a sensor, (ii) amount of processing which is required for extraction of each spatio-temporal features, (iii) commercial establishment of platforms for being use in wildlife monitoring, and (iv) invasiveness with respect to their effect on the animal under study. Under the processing required column, we mark a technology with Low when low amount of computation is needed on the device to extract a spatio-temporal feature, and High is used otherwise. It can be seen that although the technologies used for the Eulerian approach can provide more information, extraction of these information requires considerable amount of offline data analysis.

### 5.3. Comparison of Technologies Based on the Subject of Study

Table 3 summarizes the aforementioned technologies based on their usage in different wildlife monitoring studies. For each technology, we provide a number of available studies performed on extraction of data from a specific type of species along with the feature which has been collected. It should be mentioned that the references which are given under each animal type are only those which have been included in this paper. It is also worth mentioning that not all of these technologies have been used in combination with wireless sensor networks. However they have the potential to be used in such networks. If a field under a species type is marked with (-), it means that the corresponding technology is not popularly used for studying that species.

It can clearly be concluded from Table 3 that not all technologies are equally appropriate and used for all types of species. Almost all technologies have been used for mammals while a very few technologies have been tested and used for fish and amphibians. Not many of these sensing technologies have been used in combination with wireless sensor networks. This introduces a whole new set of opportunities for the use of wireless sensor networks for wild life monitoring research.

## 6. Conclusions and Future Directions

Wireless sensor networks provide additional advantages over previous telemetry methods in collecting spatio-temporal data, which make them suitable for various wildlife monitoring applications. Two types of movement modeling are possible using these networks (*i.e.*, Lagrangian, and Eulerian). Collecting spatio-temporal data with wireless sensor networks especially for the Eulerian approach has various unexplored domains, though research in this domain are few and relatively sparse. To provide suitable outcome for ecological modeling, further improvements are still needed in terms of software (for extracting spatio-temporal features) as well as hardware (for sensing). Different schemes in the domain of human sensing have not yet been applied to wildlife monitoring. For instance, gait biometric, a well-explored biometric in human-sensing, has not been used in wildlife monitoring projects. Gait pattern has the potential to be detected with different sensors (radar, seismic, visual, and acoustic) for extracting species type, identity or maybe physiological state information that it conveys. Furthermore, in some domains such as chemical sensing the technology still has to improve to be able to provide spatio-temporal data. For instance, although there is evidence that different physiological states may be measured through chemical change in individuals, the technology that is usable in conjunction with wireless sensor networks remains undeveloped.

## Figures and Tables

**Table 1.** Comparison of sensing technologies in terms of spatio-temporal features they provide.

	**Technology**	**Presence**	**Species Type**	**Identity**	**Location**	**Invasive**	**Type**
**Eulerian**	Radar	√	√	√	√	-	Active
	Geophones	√	√	√	√	-	Passive
	Microphones	√	√	√	√	-	Passive
	Thermal cameras	√	√	-	√	-	Passive
	PIR	√ (only in motion)	-	-	-	-	Passive
	Thermometers	√	√	-	-	-	Passive
	Electronic noses	-	-	-	-	-	Passive
	Cameras	√	√	√	√	-	Passive

**Lagrangian**	Passive RFID tags	√	√	√	√	√	Passive
	Active RFID tags	√	√	√	√	√	Active
	GPS	√	√	√	√	√	Active
	Inertial sensors	√	√	√	√	√	Passive
	Radio transmitters	√	√	√	√	√	Active

**Table 2.** Comparison of sensing technologies based on a number of performance metrics. P (presence), I (Identity), S (species type), L (location).

	**Technology**	**Outdoor Disruptions**	**Processing Required**				**Commercially Established**	**Invasive**

			**P**	**S**	**I**	**L**		
**Eulerian**	Radar	Smoke, dust, humidity	Low	High	High	High	-	-

	Geophones	Cultural noise	High	High	High	High	-	-

	Microphones	Wind, background acoustic noise	Low	High	High	High	√	-

	Thermal cameras	Smoke, dust, humidity	High	High	High	High	√	-
	
	PIR		Low	-	-	-	√	-
	
	Thermometers		High	High	-	-	√	-

	Electronic noses	Humidity, air quality, wind	-	-	-	-	-	-

	Cameras	Unsuitable lighting conditions	High	High	High	High	√	-

**Lagrangian**	Passive RFID tags	-	Low	Low	Low	Low	√	√

	Active RFID tags	-	Low	Low	Low	Low	√	√

	GPS	Cloud cover, heavy foliage, indoors	Low	Low	Low	Low	√	√

	Inertial sensors	Metal objects, external magnetic field and gravity	Low	Low	Low	Low	√	√

	Radio transmitters	-	Low	Low	Low	Low	√	√

**Table 3.** Summary of the technological solutions with respect to the studied animal.

**Technology**	**Animals**					**Humans**
	**Mammals**	**Birds**	**Amphibians**	**Reptiles**	**Fish**	
Radar, ultrasound	Dog and horse [18] (Species type 2007)	[19] (Presence 2008) [20] (Track 2010) [21] (Track 2005)	-	-	[22] (Species type 2003)	[16] (Identity 2008) [14] (Identity 2002) [18] (Presence 2007)
Camera	Lion [36] (Track 2006) Zebra [30] (Identity 2011) Cows [33] (Identitiy 2008) Quadrupeds [32] (Species type 2001) Rats [39] (Behavior 2005 Track) Wild animal [137] (Track 2010)	[34] (Tracking, 2002)	-	Snakes [40] Behavior analysis 2007)	-	[138] (Identitiy 2004) [139] (Identitiy 2004) [140] (Identity 2004)
Infrared technologies	Cows [50] (Disease 2011) [42] (Stress2008) Quoll [44] (Presence 2004)Zoo mammals [49] (Disease 2005)	Ostrich [49] (Disease 2005)	-	Lizzard [141] (Temperature model 2012)	-	[46] (Presence 2010) [47] (Identification 2007)
E-nose	Livestock [59] (Farm odors 2009)	-	-	-	-	[60] (Identification 2009)
Geophone	Quadrupeds [78] (Presence 2009)Elephants and large mammals [92] (Species 2005) Mole rat [93] (Seismic communication)	-	-	-	-	[85] (Presence 2010) [88] (Presence 2007) [89] (Presence 2003) [86] (Tracking 2001)
Microphone	Lycaon pictus [73] (Identity 2005)	Crane [72] (Identity 2000) Eagle owl [74](Identity 2008) Ant-thrushes [75] (Identity 2011)	Cane-toad [69] (Species 2004)[142] (Species 2005) Anurans [67] (Species type 2002)	-	-	[76] (Presence 2007) [77] (Idnetity 2007)
RFID	Badgers [98] (Presence 2010) Farm animals [99] (Presence 2006)	Tern [95] (Presence 1997)	Salamanders [97] (Presence 2007)	Corn snake [96] (Presence 2000)	[104] (Presence 2000)	[100] (Presence 2005)
GPS	Livestock [126] (Track 2004) [127] (Track 2009)Zebra [123] (Track 2002)Caprocorn [143] (Track 2012) Mountain lions [124] (Track 2011)	Migratory birds [113] (Track 2009) [112] (Track 2009) [144] (Track 2008)	-	-	-	[145] (Track 2009)
Inertial sensors	Rats [130](Track 2008) [131](Track 2009) Cows [134] (Activity 2008) [84] (Activity 2008)Different species [133] (Actvitiy 2008)	-	-	-	-	[128] (Tracking 2001) [129] (Tracking 2005)
Radio transmitters	Cows [135] (Tracking 2010)	-	-	-	-	-
